# Therapeutic Efficacy of Albendazole in Neonatal Calves Naturally Infected with *Cryptosporidium parvum*

**DOI:** 10.3390/pathogens15080854

**Published:** 2026-08-16

**Authors:** Okan Bayrak, Mehmet Can Ulucesme, Meryem Toprak Tuncer, Munir Aktas, Ahmet Atessahin

**Affiliations:** 1 Department of Pharmacology and Toxicology, Faculty of Veterinary Medicine, Fırat University, Elazığ 23119, Türkiyem.toprak@firat.edu.tr (M.T.T.); 2 Department of Parasitology, Faculty of Veterinary Medicine, Fırat University, Elazığ 23119, Türkiye; mculucesme@firat.edu.tr (M.C.U.); maktas@firat.edu.tr (M.A.)

**Keywords:** neonatal calf diarrhea, cryptosporidiosis, treatment, benzimidazole, oocyst shedding

## Abstract

Cryptosporidiosis is a significant zoonotic disease caused by *Cryptosporidium* species, primarily *Cryptosporidium parvum*, leading to serious economic losses in neonatal calves; the absence of an approved vaccine or fully effective treatment necessitates alternative therapeutic approaches. This study evaluated the therapeutic efficacy of albendazole (20 mg/kg, orally, 5 days) in naturally infected neonatal calves, comparing it to paromomycin (100 mg/kg, orally, 5 days) and an albendazole + paromomycin combination. Forty-eight Simmental calves (36 naturally infected, mean age of 14 days, and 12 healthy controls) were included. Infection was confirmed by rapid immunochromatographic tests, carbol fuchsin staining, nested PCR, and RFLP, identifying *C. parvum* as the causative agent. Fecal and blood samples were collected on days 0, 3, 7, 10, 20, and 30, alongside clinical evaluations and live weight measurements. Oocyst shedding declined significantly over the study period in all three treatment groups, with the paromomycin and albendazole + paromomycin groups showing a numerically earlier decline than the albendazole group; however, no statistically significant differences among the three treatment groups were detected at any individual sampling day. Fecal scores paralleled oocyst shedding patterns. No significant differences were detected among treatment groups in hematological, biochemical, or hepatorenal safety parameters. Albendazole demonstrated efficacy in reducing oocyst shedding in *Cryptosporidium* infection, suggesting it may serve as a viable alternative treatment option in field conditions.

## 1. Introduction

Cryptosporidiosis is a significant zoonotic disease affecting human and animal health worldwide, caused by *Cryptosporidium* species, which are obligate intracellular protozoa belonging to the phylum Apicomplexa [1,2,3]. This disease, which is among the primary etiological causes of enteritis, particularly in neonatal calves, is transmitted via the fecal-oral route [4,5]. *Cryptosporidium parvum* is the species most closely associated with clinical cryptosporidiosis in newborn calves; it invades intestinal epithelial cells, leading to villus shortening, fusion, and mucosal erosion, as well as hypertrophy of the small intestinal crypts. This pathological process leads to digestive dysfunction, increased intestinal permeability, watery diarrhea, dehydration, and in severe cases, death [6,7,8].

Studies conducted in various countries have reported that the prevalence of cryptosporidiosis in cattle ranges widely from 11.7% to 78% [5,9,10,11,12,13]. In Türkiye as well, studies conducted in different provinces have reported the prevalence of *Cryptosporidium* spp. in calves with diarrhea ranging from 5.94% to 63.3%, while molecular studies have identified rates ranging from 13.6% to 58.2% [14,15,16,17,18]. The economic impact of the disease extends beyond high morbidity and mortality rates to include calf deaths, treatment costs, stunted growth, and a slowdown in genetic progress within the herd [19].

Of the numerous antimicrobials and antiparasitic agents studied to date for the treatment of cryptosporidiosis, only halofuginone lactate has been licensed in Europe for the control of the disease in ruminants, and its efficacy is controversial [20]. In the United States, there is no approved treatment for cryptosporidiosis [21,22]. Paromomycin is the most frequently used pharmacological agent in the field for this purpose. Nitazoxanide, azithromycin, and various experimental compounds show limited efficacy [23,24,25]. Filling this pharmacological gap necessitates the development of new and cost-effective treatment protocols.

Albendazole is a broad-spectrum anthelmintic drug belonging to the benzimidazole group. Its primary mechanism of action involves inhibiting tubulin polymerization, thereby disrupting the cellular metabolism and replication of parasites [26,27]. In veterinary medicine, albendazole is widely used for the treatment and control of gastrointestinal nematodes such as *Haemonchus contortus*, *Ostertagia ostertagi*, *Trichostrongylus* spp., *Cooperia* spp., and *Nematodirus* spp., as well as lung parasites such as *Dictyocaulus* spp., primarily in ruminants including cattle, sheep, and goats [28]. In ruminants, albendazole is also considered an effective antiparasitic agent against liver fluke infections caused by Fasciola hepatica; it demonstrates efficacy against cestodes such as *Moniezia* spp. and is among the most used broad-spectrum anthelmintics in the livestock industry [29,30]. Its low cost, wide availability, and long-standing use in veterinary medicine make albendazole a potential alternative. However, the lack of clinical data on the therapeutic efficacy of albendazole in *Cryptosporidium* infections necessitates further investigation of this gap [31].

The aim of this study was to compare the therapeutic efficacy of albendazole (20 mg/kg, oral, 5 days) with that of paromomycin (100 mg/kg, oral, 5 days) in neonatal calves infected with natural *C. parvum*, and to evaluate whether the albendazole + paromomycin combination confers additional benefit over either agent alone. To this purpose, hematological, biochemical, clinical, and vital parameters, as well as oocyst shedding processes, were examined in the animals; for the diagnosis of infection, carbol fuchsin staining, immunochromatographic rapid diagnostic kit, nested polymerase chain reaction (PCR), restriction fragment length polymorphism (RFLP), and gene sequencing methods were used.

## 2. Materials and Methods

### 2.1. Ethical Approval and Work Location

This study was supported by the TÜBİTAK 1001 Project Support Program under project number 223O148. The research was conducted with the approval of the Bingöl University Local Ethics Committee for Animal Experiments (protocol code 2023/02-02/01, 31 March 2023), and all procedures were carried out in accordance with animal welfare principles. The study was conducted at the Bingöl Integrated Facilities, which operate within the scope of the Sütaş Eastern-Southeastern Anatolia Dairy Project; all calves in the experimental and control groups were subjected to the same care and feeding conditions. Because the study was performed under commercial field conditions on clinically affected neonatal calves, the ethics approval did not permit withholding treatment from diarrhoeic calves. Consequently, an infected-untreated group could not be included.

### 2.2. Animal Materials and Housing Conditions

The study included a total of 48 animals: 36 neonatal calves of the Simmental breed with diarrhea, naturally infected with *Cryptosporidium* spp., and 12 healthy control calves. Calves were reared from day 5 of life under the farm’s standard management; diarrhoea developed and treatment was initiated at a mean age of 14.14 ± 1.42 days (range 12–16 days; median 14 days), corresponding to the reported peak of natural oocyst shedding in neonatal calves [7]. All animals were selected from those receiving the same care and feeding conditions in accordance with the farm’s routine practices.

All calves included in the study were housed in individual care pens with straw bedding and dry flooring. All animals were provided with ad libitum drinking water and feed. Water and feed buckets were cleaned daily at the farm’s washing station. To prevent potential reinfection, equipment used for the calves and the care pens were regularly disinfected with Virkon^®^-S (LANXESS, Sudbury, UK). Employees involved in medication administration and clinical evaluations wore disposable protective clothing, caps, masks, boot covers, and gloves. All medical supplies used were disposed of in accordance with medical waste regulations. The calves were fed twice daily (at 8:00 a.m. and 6:00 p.m.) with 2.5 L of milk replacer per feeding. Throughout the study period, all animal care, feeding and hygiene practices were conducted in accordance with the farm’s standard procedures.

All pregnant cows and heifers on the farm were administered two doses of a combined vaccine (Kolibin^®^ RC Neo, Bioveta, Ivanovice na Hané, Czech Republic) against rotavirus, coronavirus, and *Escherichia coli*, an anti-clostridial vaccine (Coglavax^®^, Ceva Santé Animale, Libourne, France), and a mastitis vaccine (Startvac^®^, HIPRA, Amer, Girona, Spain) two months and one month prior to calving, respectively. All calves were ensured to receive at least 3 L of colostrum within the first 1–2 h after birth, for a total of approximately 6 L per day. Additionally, all calves were administered a combined antiserum Multisera^®^ (Ata Fen, Kemalpaşa, İzmir, Türkiye) against *E. coli*, *Salmonella typhimurium*, *S. dublin*, *Trueperella pyogenes*, *Clostridium perfringens*, *Pasteurella multocida*, and *Mannheimia haemolytica* within the first 24 h following birth. All calves that reached one week of age were administered an intranasal vaccine (Rispoval^®^ IntraNasal, Zoetis, Parsippany, NJ, USA) against Parainfluenza type 3 (PI3) and Bovine Respiratory Syncytial Virus (BRSV).

Calves exhibiting symptoms of diarrhea caused by coronavirus, rotavirus, or other bacterial or parasitic agents, as well as respiratory infections and signs of infections affecting other systems, were excluded from the study. Additionally, during the study period, no suspected clinical signs consistent with cryptosporidiosis were reported among the farmer or farm workers who were in contact with the calves.

### 2.3. Formation of Groups and Treatment Protocols

Calves meeting the case definition were enrolled consecutively upon detection during routine daily farm health checks and were allocated to one of the three treatment groups by simple randomisation using a pre-generated allocation sequence. Because the study was conducted under commercial field conditions, enrolment occurred as clinical cases arose over the calving season. The resulting age at treatment initiation was homogeneous across groups (albendazole 14.50 ± 1.68 days; paromomycin 13.67 ± 1.44 days; albendazole + paromomycin 14.25 ± 1.06 days) and did not differ significantly among groups (*p* = 0.345).

A total of 48 neonatal calves were included in the study; they were divided into four groups: a control group (*n* = 12) and three treatment groups (*n* = 12 each) consisting of calves with confirmed *Cryptosporidium* spp. infection. The inclusion criterion was the presence of at least 1.2 × 10^5^ *Cryptosporidium* spp. oocysts per gram of feces. This threshold was applied to ensure enrolment of animals with a confirmed, clinically relevant patent infection and to exclude incidental low-level shedders whose diarrhoea might be attributable to other enteropathogens; it was based on clinically significant high oocyst loads [12] and inclusion ranges used in field treatment studies [32]. Observed baseline loads (≈1.5 × 10^8^ oocysts/g) were three orders of magnitude above this threshold, indicating that all enrolled calves were heavy shedders. Enrolled calves presented with mild-to-moderate, uncomplicated diarrhea with preserved suckling reflex and normal mental status. The study groups and treatment protocols applied are outlined below.

Group 1—Control (*n* = 12): Consisted of clinically healthy calves and did not receive any medication.

Group 2—Albendazole (*n* = 12): Calves diagnosed with cryptosporidiosis were administered albendazole (Vetalben^®^, Vetaş, Tekirdağ, Türkiye, 10%, oral solution) at a dose of 20 mg/kg, once daily for 5 days, via the oral route.

Group 3—Paromomycin (*n* = 12): Calves diagnosed with cryptosporidiosis were administered paromomycin (Gabbrocol^®^, Ceva Santé Animale, Libourne, France) at a dose of 100 mg/kg, once daily for 5 days via the oral route [33].

Group 4—Albendazole + Paromomycin (*n* = 12): Calves diagnosed with cryptosporidiosis were administered albendazole (20 mg/kg) and paromomycin (100 mg/kg) together, once daily for 5 days, via the oral route.

Should any calf in any group have developed signs of significant dehydration or a weakened suckling reflex, parenteral fluid and electrolyte therapy containing isotonic sodium chloride (0.9% NaCl) and sodium bicarbonate (2 mL/kg, Carbotek^®^, Teknovet, İstanbul, Türkiye) was available as a contingency, continued on subsequent days according to the degree of dehydration. This supportive protocol was defined a priori for animal welfare reasons and does not describe the clinical phenotype at enrolment, which was mild to moderate.

### 2.4. Sample Collection

Fecal samples were collected from calves via rectal palpation or spontaneous defecation for the purpose of determining daily fecal scores and fecal consistency, as well as for DNA extraction. To prevent contamination during the fecal collection process, separate single-use sterile gloves were used for each calf. All samples were obtained from fresh feces. Fecal samples were collected on days 0, 3, 7, 10, 20, and 30 in accordance with the study protocol. The collected fecal samples were placed in sterile containers until the analysis procedures were completed and stored at +4 °C for short-term storage.

Blood samples were collected from all calves via the jugular vein on days 0, 3, 7, 10, 20 and 30 using Vacutainer needles in accordance with aseptic and antiseptic protocols. During each sampling, 2 mL of blood was collected into an EDTA-containing tube and 5 mL into an anticoagulant-free gel serum tube. Blood samples collected in anticoagulant-free tubes were centrifuged at 3000 rpm 5 min after clotting was complete. The resulting serum samples were transferred to three separate microcentrifuge tubes per sample and stored at −20 °C until laboratory analyses were performed.

Complete blood count (CBC) tests; white blood cell (WBC), red blood cell (RBC), hemoglobin (HGB), hematocrit (HCT), platelet (PLT), lymphocyte (LYM), neutrophil (NEU), monocyte (MON), eosinophils (EOS), mean corpuscular volume (MCV), mean corpuscular hemoglobin (MCH), mean corpuscular hemoglobin concentration (MCHC), red blood cell distribution width-coefficient of variation (RDW-CV), red blood cell distribution width-standard deviation (RDW-SD), mean platelet volume (MPV), platelet distribution width (PDW), and plateletcrit (PCT) were determined at the Fırat University Animal Hospital Laboratory using an automated hematology analyzer (Mindray BC-2800 Vet, Shenzhen, China).

Biochemical analyses the parameters aspartate aminotransferase (AST), gamma-glutamyl transferase (GGT), total protein (TP), albumin (ALB), blood urea nitrogen (BUN), urea (UREA), and creatinine (CREAT) were evaluated using an automatic biochemistry analyzer (Mindray BS-120, Shenzhen, China).

### 2.5. Clinical Examination and Fecal Scoring

Calves with diarrhea caused by *Cryptosporidium* spp. were subjected to daily clinical examinations from the first day of infection (day 0) through day 10. During daily clinical examinations, the following parameters were recorded: body temperature (°C, measured with a rectal thermometer), respiratory rate (breaths/minute), heart rate (beats/minute), suckling reflex score (0–3), fecal clinical appearance score (0–3), and mental status score (0–3). All clinical examinations, medication administrations, and sample collection procedures were conducted daily during the same time window (09:00–10:00) to ensure standardization [34].

### 2.6. Mental Status, Sucking Reflex and Fecal Scoring

The criteria reported by Evci and Sentürk [35] and the Wisconsin-Madison calf health scoring system [36] were used to assess mental status. The clinical appearance scores of the calves’ feces were calculated according to the 4-point scoring system defined by Larson et al. [37] (Table 1).

### 2.7. Live Weight Measurements

All calves included in the study were weighed on days 0, 5, 10 and 30. Weighings were performed using a calibrated digital livestock scale during the same time window on each measurement day and prior to feeding; the obtained values were recorded in kilograms. Care was taken to ensure the calves were calm during the measurement, and the weighing procedures were completed quickly to minimize measurement errors caused by movement. Because the healthy control calves were expected to be heavier at enrolment, growth was additionally expressed as the within-animal percentage change from day 1, with each calf serving as its own baseline, so that between-group comparisons of weight gain are not biased by differing starting weights.

### 2.8. Diagnostic Methods for Cryptosporidium spp.

To enable definitive species identification in the animals included in the study, an immunochromatographic rapid test kit, carbol fuchsin staining method and PCR techniques were applied simultaneously. The combined use of these three different methods was chosen to enhance diagnostic reliability.

### 2.9. Rapid Immunochromatographic Diagnostic Test Kit

The BIO K 288 Rainbow Calf Scour Diagnostic Test 5-pack rapid diagnostic kit (Bio-X Diagnostic, Rochefort, Belgium) was applied to fresh fecal samples collected via rectal swab or spontaneous defecation from calves with diarrhea suspected of having cryptosporidiosis. This test kit detects antigens specific to pathogens using a rapid immunochromatographic method on a strip membrane; each strip contains a layer of monoclonal antibodies specific to the target pathogen. The kit can simultaneously detect the presence of infectious agents such as *Cryptosporidium* spp., rotavirus, coronavirus, *C. perfringens*, and *E. coli* F5 (K99). Calves testing positive for these agents were excluded from the study due to suspected mixed infections. The test was administered to all groups on days 0, 3, 7, 10, 20 and 30 during the study period.

### 2.10. Carbol Fuchsin Staining Method

The carbol fuchsin staining method was used to confirm the diagnosis in calves that tested positive for cryptosporidiosis using an immunochromatographic rapid test kit. Fecal samples collected from the calves were suspended by adding an equal volume of 2.5% potassium dichromate (K_2_Cr_2_O_7_) solution and stored at +4 °C during the analysis period [38]. Using a pipette, 50 µL of the homogenized fecal sample was transferred onto a slide that had been cleaned with an ether-alcohol mixture to remove grease. An equal amount (50 µL) of carbol fuchsin stain (A.D.R. Advanced Diagnostics & Research, Cat. No: 150008-2, Mediko Kimya, İstanbul, Türkiye) was added to the sample, and a thin fecal smear was prepared using the edge of the coverslip. Immediately after the prepared smear was air-dried, a drop of immersion oil was added, the coverslip was placed over it, and the sample was examined under a light microscope at ×100 magnification for the presence of *Cryptosporidium* oocysts [39].

### 2.11. Oocyst Count

The counting of *Cryptosporidium* spp. oocysts was performed on the same day the fecal samples were collected. For this purpose, smears prepared from 1 g of fecal sample using the carbol fuchsin staining technique were examined under a light microscope at ×100 magnification using the method described by Atwal et al. [40], and oocyst counts were performed. The inclusion criterion for the study was the presence of at least 1.2 × 10^5^ *Cryptosporidium* spp. oocysts per 1 g of stool. The initial oocyst count was performed prior to treatment (day 0); to evaluate the efficacy of the treatment, the same protocol was repeated on days 3, 7, 10, 20 and 30.

### 2.12. DNA Isolation

Fresh fecal samples from calves with cryptosporidiosis confirmed via carbol fuchsin staining were sent to the Department of Parasitology at Fırat University Faculty of Veterinary Medicine for DNA isolation via the cold chain. For genomic DNA isolation, 200 mg of feces was weighed from each sample, and the process was performed using a commercial stool DNA extraction kit (Exgene™ Stool DNA mini–Extraction Kit, GeneALL Biotechnology, Seoul, Republic of Korea) in accordance with the steps specified in the kit protocol. The quality and concentration of the isolated DNA were verified using a spectrophotometer (NanoDrop^®^ ND-2000 UV/Vis Spectrophotometer, Thermo Fisher Scientific Inc., Wilmington, DE, USA), and the resulting genomic DNA samples were stored at −20 °C until subjected to PCR analysis.

### 2.13. Nested PCR

A two-step nested PCR method was applied to DNA samples obtained for the molecular confirmation of Cryptosporidiosis infection. The small subunit ribosomal RNA (SSU rRNA) gene, which is considered the standard for the phylogenetic differentiation of *Cryptosporidium* species, was selected as the target gene. In the first stage of PCR, the CryptoF (5′-TTCTAGAGCTAATACATGCG-3′) and CryptoR (5′-CCCATTTCCTTCGAAACAGGA-3′) primers were used to amplify a 1325 bp DNA fragment from the SSU rRNA gene region of *Cryptosporidium* species. In the nested PCR stage, the primers CryptoNF (5′-GGAAGGGTTGTATTTATTAGATAAAG-3′) and CryptoNR (5′-AAGGAGTAAGGAACAACCTCCA-3′) were used to amplify a DNA fragment approximately 826–864 bp in length [41].

Reactions for both PCR steps were prepared in a total volume of 25 µL. The reaction mixture consists of 2.5 µL PCR buffer, 2.5 µL MgCl_2_ (25 mM), 125 µM of each deoxynucleotide triphosphate (dNTP; dATP, dCTP, dGTP, dTTP), 0.1 µL (1,25 U) Taq DNA polymerase, 1.25 µL of each primer (from a stock concentration of 20 pmol/µL), and 2.5 µL of template DNA. The same reaction components were used in the nested PCR step; 1 µL of the first PCR product was used as the template. Positive and negative control DNA samples from the laboratory stock were included in each PCR run. Amplification was performed using a thermal cycler (SensoQuest, Labcycler Gradient, Göttingen, Germany). A 5 µL aliquot of the amplification products obtained from the PCR was run on a 1.4% agarose gel (in Tris-Borate-EDTA buffer) alongside a 100 bp DNA ladder H3 RTU (DM003-R500, GeneDireX^®^, BIO-HELIX Co., Ltd., New Taipei City, Taiwan) spanning 100–3000 bp, which resolves both the ~1325 bp primary product and the ~826–864 bp nested product unambiguously. After the agarose gel electrophoresis was completed, the gel was visualized under UV light and photographed using a gel imaging system (Quantum Vilber Lourmat, Collégien, France).

### 2.14. Species Identification Using Restriction Fragment Length Polymorphism (RFLP) Analysis

Following the amplification of the target DNA sequence via nested PCR, RFLP analysis was performed to identify *Cryptosporidium* species. Two different restriction endonuclease enzymes were used. The *SspI* restriction enzyme allows for the general identification of *Cryptosporidium* spp., and this enzyme can distinguish between *C. parvum*, *C. bovis* and *C. ryanae* [42,43]. The characteristic restriction band sizes for *C. parvum* are 449 bp + 267 bp + 108 bp. The *MboII* restriction enzyme is specifically used to distinguish *C. parvum* from other species. The expected band size for *C. parvum* is 771 bp.

In RFLP analyses, New England Biolabs rCutSmart (Ipswich, MA, USA) *SspI* and *MboII* enzymes were used, and the manufacturer’s kit protocols were followed. RFLP reactions were prepared in a total volume of 20 μL, separately for each enzyme, and after being incubated in a water bath at 37 °C for 2 h, they were subjected to 1 h of electrophoresis on a 3% agarose gel. Following electrophoresis, the restriction profiles were evaluated under UV light using a gel imaging system. RFLP analysis was used to identify the *Cryptosporidium* species *C. parvum*, *C. bovis* and *C. ryanae*, which cause cryptosporidiosis, to investigate the presence of mixed infections.

### 2.15. DNA Sequencing

To molecularly validate the RFLP analysis results, nested PCR products obtained with CryptoNF/CryptoNR primers amplifying the SSU rRNA gene region were sent to a commercial firm (BM-Labosis, Ankara, Türkiye) for bidirectional sequence analysis. Two PCR products, selected to represent each group from samples identified according to PCR and RFLP results, were included in the sequence analysis. Raw nucleotide sequences were visualized and evaluated using FinchTV 1.4.0. The gene sequences obtained from the sequence analysis were submitted to the EMBL/GenBank database. The obtained sequences were compared with other isolates registered in GenBank using the BLASTN Basic Local Alignment Search Tool (BLAST) program on the National Center for Biotechnology Information (NCBI) website (https://blast.ncbi.nlm.nih.gov/Blast.cgi).

### 2.16. Statistical Analysis

Data normality was assessed using the Shapiro–Wilk test, homogeneity of variances using the Levene test, and the sphericity assumption using Mauchly’s W test. For the hematological, biochemical and vital parameters, which conformed to the assumptions of normality, repeated measures analysis of variance (ANOVA) was used to analyze the data, and the findings are expressed as the arithmetic mean ± standard error ($\overset{-}{X}$ ± SE). Fecal oocyst counts, in contrast, did not conform to a normal distribution, and the standard deviation associated with these counts was disproportionately large relative to the arithmetic mean; oocyst data are therefore reported as median (range: minimum-maximum) and were analyzed using non-parametric tests. The effect of time within each treatment group was assessed using the Friedman test, and comparisons among treatment groups at each sampling day were performed using the Kruskal–Wallis test. Changes in the proportion of oocyst-positive calves over time were examined using Kaplan–Meier survival analysis; the oocyst clearance days in the experimental groups during the follow-up period were illustrated using survival curves. Survival curves visually illustrate the oocyst shedding duration between groups. In this study, the *p*-value of <0.05 was considered statistically significant. Analyses of hematological, biochemical, vital and live-weight data were performed using The Jamovi Project v2.3 software package; the non-parametric analyses of oocyst count (Friedman and Kruskal–Wallis tests) were performed in R (version 4.5.3; R Core Team 2025) using the lmerTest, emmeans and stats packages. GraphPad Prism (v8; GraphPad Software, San Diego, CA, USA) software was used for plotting the graphs.

## 3. Results

Prior to treatment, the three infected groups were comparable. Age at treatment initiation did not differ among groups (*p* = 0.345), and baseline (day 0) oocyst load did not differ significantly among the albendazole, paromomycin and albendazole + paromomycin groups. At enrolment, all infected calves had a fecal score of 2, a suckling reflex score of 3, and a mental status score of 0, whereas all control calves had a fecal score of 0. Thus, the groups did not differ in age, parasite burden or clinical severity before treatment.

### 3.1. Hematological and Biochemical Parameters

Among white blood cell parameters, no differences were detected between groups for WBC (*p*_group_ = 0.356), NEU (*p*_group_ = 0.583) or LYM (*p*_group_ = 0.500). Significant group differences were observed for monocytes (MON, *p*_group_ = 0.019; highest in the paromomycin group, lowest in the albendazole group) and eosinophils (EOS, *p*_group_ = 0.006; highest in the control group). Measurement day significantly affected all leukocyte parameters (*p*_measurement_ < 0.001 for WBC, LYM, MON and EOS; *p*_measurement_ = 0.009 for NEU), with WBC and LYM peaking on days 1 and 30, reflecting the acute phase of infection and the recovery period, respectively. EOS values rose significantly on days 20 and 30 in all groups and were lowest on day 7; on day 30, EOS in the treatment groups exceeded that of controls.

Among red blood cell parameters, no group differences were found for RBC, HGB, HCT, MCV (*p*_group_ = 0.051) or MCH (*p*_group_ = 0.232), although all showed significant day effects (*p*_measurement_ < 0.001) and group × time interactions. MCHC differed significantly between groups (highest in the paromomycin group, lowest in controls; peak on day 3), as did RDW-CV and RDW-SD (both highest in the albendazole + paromomycin group, lowest in controls), with RDW values declining to their lowest levels by day 30. Among platelet parameters, PLT (*p*_group_ = 0.165), MPV (*p*_group_ = 0.836), PDW (*p*_group_ = 0.853) and PCT (*p*_group_ = 0.097) showed no group differences; PLT varied significantly by day (highest on day 1, 695.20 ± 27.90 × 10^9^/L; lowest on day 20, 506.44 ± 29.50 × 10^9^/L).

Among biochemical analytes, no group differences were detected for TP, UREA, GGT, BUN, or CREAT. ALB differed significantly between groups: ALB was lower in all treatment groups than in controls (lowest in the paromomycin group), with the lowest on day 3. AST activity was higher in controls than in the treatment groups and peaked on day 30 in all groups. GGT activity was elevated in all groups on day 0 and declined progressively thereafter, consistent with the physiological decline following intestinal absorption of colostral GGT [44,45]. Critically, CREAT, BUN, and UREA in the albendazole group remained within age-specific reference ranges and did not differ from controls (*p*_group_ = 0.406; *p*_measurement_ = 0.197), indicating that albendazole was well tolerated in neonatal calves during short-term use.

Only statistically significant findings are summarised here; the full time-course graphs for all haematological and biochemical analytes are provided as Supplementary Material (Figures S1–S6).

### 3.2. Vital Parameters

Throughout the study, body temperature in all groups remained within the physiological range of 38.5–39.5 °C; no significant trend toward increases or decrease was observed on the days of measurement. Body temperature values in the paromomycin group were generally higher than those in the other groups (overall mean: 39.16 ± 0.48 °C); however, no significant differences were detected between the control, albendazole, and albendazole + paromomycin groups. In terms of respiratory rate, lower values were recorded in the control group on all measurement days (overall average: 36.55 ± 4.95/min); it was determined that the average respiratory rates in the albendazole and paromomycin groups showed wide variation, and lower values were recorded on many measurement days compared to the other groups. In terms of heart rate, values in the control and albendazole + paromomycin groups ranged from 90 to 103 beats/min, whereas heart rates in the albendazole and paromomycin groups were observed to be lower and were measured within the range of 75–82 beats/min on most measurement days. All values remained within clinical physiological limits.

**Figure 1:**
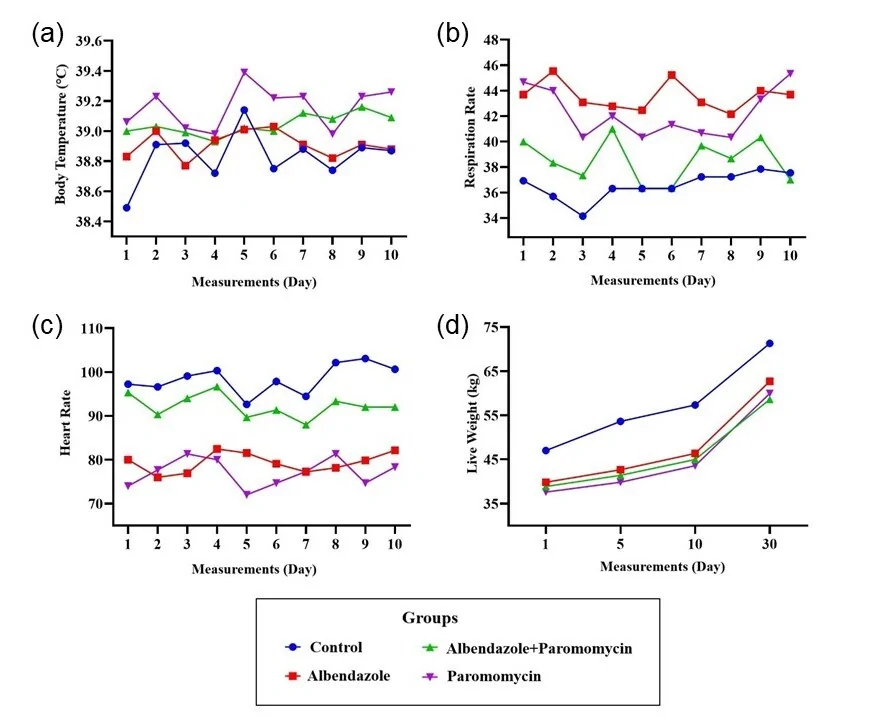
Line graphs showing changes in vital parameters and live weight across study days: (**a**) body temperature; (**b**) respiratory rate; (**c**) heart rate; (**d**) live weight.

In terms of live weight, calves in the healthy control group achieved statistically significantly higher live weight values compared to the treatment groups on all measurement days (*p* < 0.001). This is expected, as control calves were clinically healthy and non-diarrhoeic at enrolment. When growth was expressed as the within-animal percentage change from day 1, however, the treatment groups closed the gap with the control group by day 30, and the percentage gains of the albendazole (+57.3%) and paromomycin (+59.5%) groups exceeded that of the control group (+51.6%). Average live weights on day 30 were measured as follows: control group 71.29 ± 5.36 kg; albendazole group 62.71 ± 4.32 kg; albendazole + paromomycin group 58.62 ± 4.58 kg; and paromomycin group 59.97 ± 7.26 kg. In comparison among treatment groups, the overall average live weight of the albendazole group was numerically higher than that of the albendazole + paromomycin and paromomycin groups (Table 2). Group-based graphs of vital parameters and live weight according to measurement days are shown in Figure 1.

### 3.3. Diagnosis of Cryptosporidium spp.

Immunochromatographic Test Kit Analysis

Fecal samples from all calves included in the study were analyzed using a rapid immunochromatographic test kit (Table 3).

In the initial sampling of calves showing signs of diarrhea, the immunochromatographic test kit detected *Cryptosporidium* spp. antigen in all 36 calves in the treatment groups, while all 12 healthy calves in the control group tested negative. In follow-up samples collected during the treatment process, it was determined that kit positivity decreased in the albendazole group between days 7 and 10, and in the paromomycin and albendazole + paromomycin groups between days 3 and 7.

Carbol fuchsin staining

**Figure 2:**
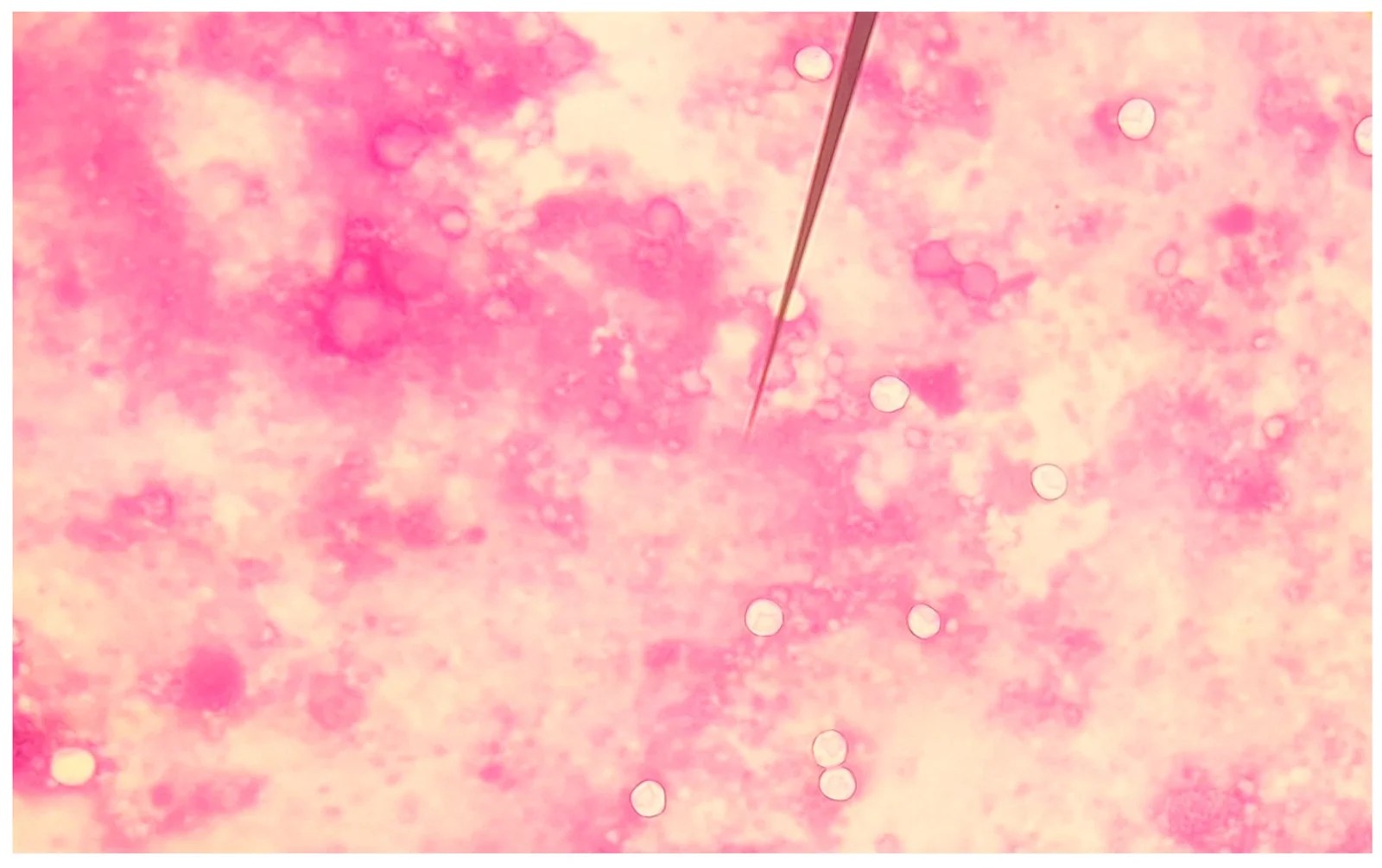
Light microscopic appearance of *Cryptosporidium parvum* oocysts in fecal smears prepared by carbol fuchsin staining (×100 objective). *C. parvum* oocysts appear pale/white against the pink-red background due to their acid-fast properties; a fine filamentous artefact visible in the field serves as a reference point adjacent to the oocysts.

Fecal smears were examined by the carbol fuchsin staining method under microscope. The oocysts appearing pale pink to colorless against the pink-red background were considered positive for *Cryptosporidium* spp. (Figure 2). In follow-up samples during the treatment process, oocyst positivity was observed to correlate with the findings of the immunochromatographic kit (Table 4).

### 3.4. Oocyst Count and Clinical Examination

To identify calves infected with *Cryptosporidium* spp., fecal samples were examined for the presence of oocysts using the carbol fuchsin staining technique; oocyst examination and counting were then performed on the same day the samples were collected to minimize potential errors in oocyst structure and count.

Because fecal oocyst counts did not follow a normal distribution (Shapiro–Wilk, W = 0.637, *p* < 0.001) and their standard deviations were disproportionately large relative to the corresponding means, oocyst data are presented as median (range: minimum-maximum) and were analyzed using non-parametric tests. Within each treatment group, the Friedman test showed a statistically highly significant effect of time on oocyst counts, confirming a clear decreasing trend in oocyst shedding from day 1 onward in all three treatment groups. Between-group (Kruskal–Wallis) comparisons were possible for days 1, 3 and 7, since oocyst counts had already reached zero in all groups by day 10; no statistically significant differences among the albendazole, paromomycin and albendazole + paromomycin groups were found at day 1. At day 10, 20 and 30, all groups reached a median oocyst count of zero, precluding further statistical comparison. Microscopic examination revealed a clear decreasing trend in the number of *Cryptosporidium* oocysts per gram of feces from day 1 to day 7 in all treatment groups. Although the paromomycin and albendazole + paromomycin groups showed a numerically earlier decline than the albendazole group, this apparent difference did not reach statistical significance at any of the compared timepoints. By days 20 and 30, no oocysts were detected in any of the treatment groups (Table 5).

**Figure 3:**
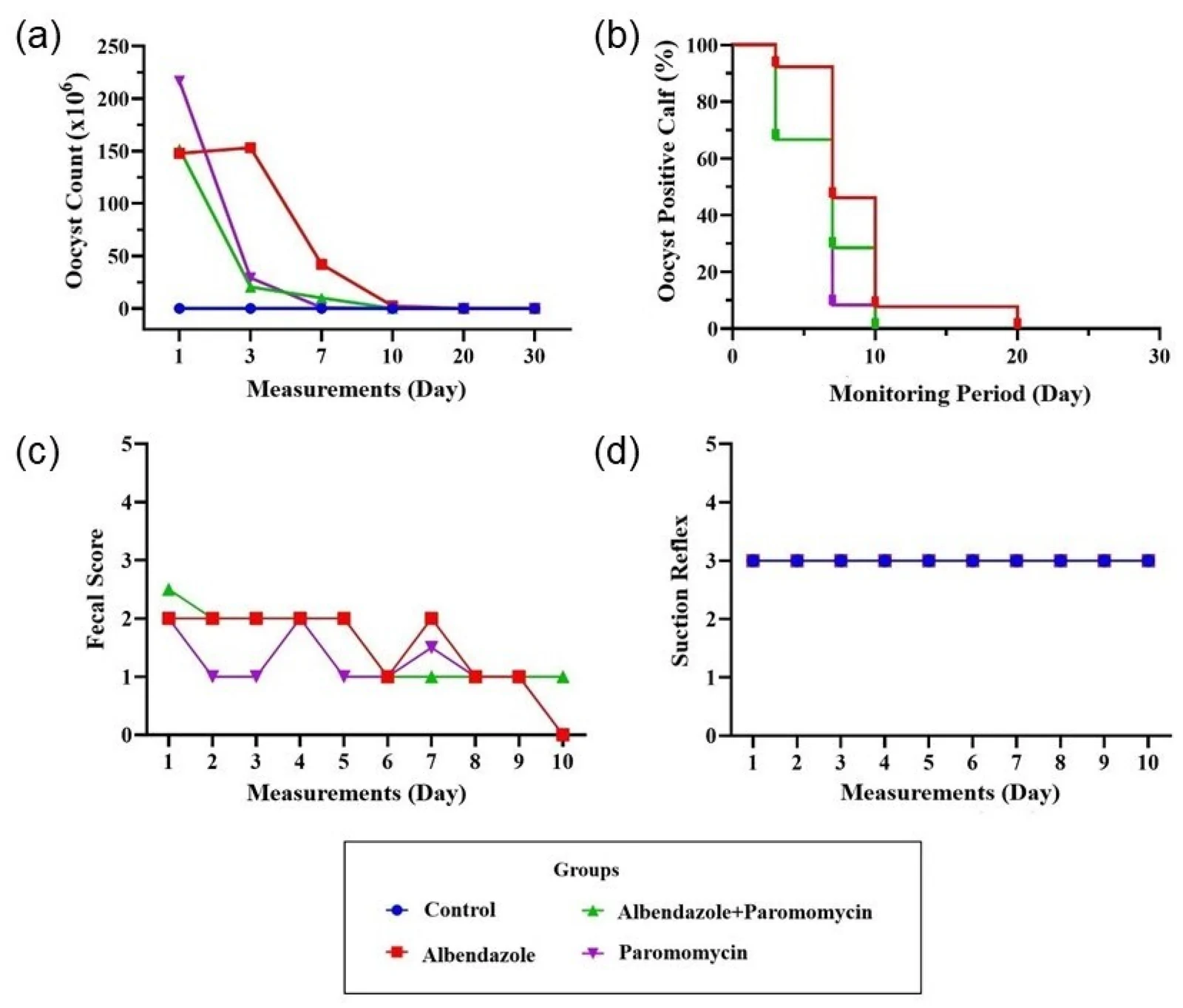
Line graphs showing changes in clinical parameters and oocyst counts across study days: (**a**) oocyst count (×10^6^); (**b**) number of oocyst-positive calves (%); (**c**) fecal score; (**d**) suction reflex. In panel (**d**), the suction reflex score remained constant at 3 in all four groups throughout the study period, resulting in complete overlap of the group lines.

In clinical examination assessments, the sucking reflex was rated as a score of 3 in all groups, including the control group, from day 1 to day 10; no differences were observed between the groups. Mental status was rated as a score of 0 in all groups and on all measurement days; no signs of depression or coma were observed in any of the calves. Regarding fecal scores, a score of 0 was recorded on all days in the control group. In the albendazole group, the fecal score was rated as 2 for the first five days; following completion of treatment, a score of 1 was recorded starting from day 5. In the albendazole + paromomycin group, the fecal score remained at level 2 for the first five days, after which it was recorded as 1. In the paromomycin group, an improvement in stool score was observed starting from day 1, and this finding was consistent with the early oocyst elimination in that group. Group-based graphs showing the number of oocysts in Gram-stained stool, fecal score, and suction reflex by measurement day are presented in Figure 3.

### 3.5. PCR and RFLP Analysis

Nested PCR was performed on genomic DNA samples obtained from the feces of a total of 48 calves from the control and treatment groups. In the first PCR step targeting the SSU rRNA gene region (CryptoF/CryptoR primers), specific amplification products of ~1325 bp were obtained, while in the nested PCR stage (CryptoNF/CryptoNR primers), products of ~826–864 bp were obtained. *Cryptosporidium* spp. infection was molecularly confirmed in all 36 calves in the treatment groups. No amplification products were detected in any of the samples from the 12 calves in the control group.

**Figure 4:**
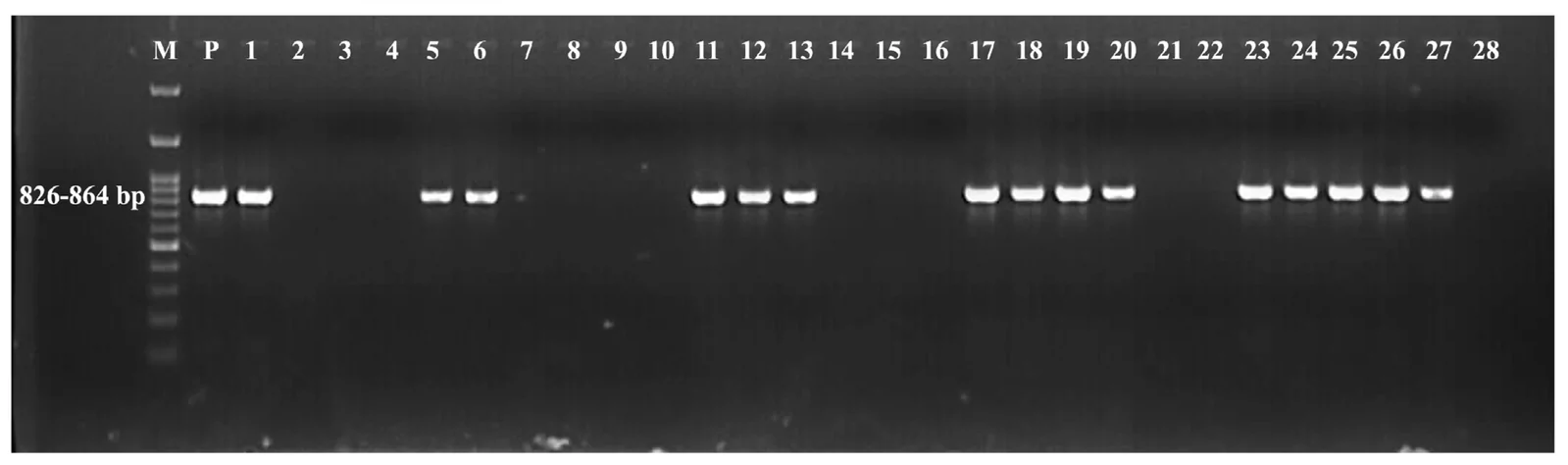
Representative agarose gel electrophoresis image of PCR amplification products from calves that remained *Cryptosporidium*-positive after treatment. P, positive control (*C. parvum*); lanes 1–4, paromomycin group (days 0, 3, 7, and 10); lanes 5–10, albendazole group (days 0, 3, 7, 10, 20, and 30); lanes 11–16, albendazole + paromomycin group (days 0, 3, 7, 10, 20, and 30); lanes 17–22 and 23–28, additional representative samples from the albendazole and albendazole + paromomycin groups, respectively, collected on the same days.

In the albendazole group, parasite DNA was detected by nested PCR in 1 of the 12 calves until day 10, in 6 calves until day 7, and in 5 calves until day 3. In the paromomycin group, parasite DNA was observed until the 10th day in 1 calf, in 11 calves, no parasite DNA was detected after the 3rd and 7th days. In the albendazole + paromomycin group, positivity was detected until the 20th day in 1 calf, until the 10th day in 1 calf, until the 7th day in 4 calves, and until the 3rd day in 5 calves; in 1 calf, no DNA was detected from the after treatment. PCR results turned negative in the late phase in all treatment groups; however, parasite DNA was detected in one calf in the albendazole + paromomycin group as late as day 20 (Figure 4).

In the RFLP analysis performed on PCR products, two different restriction enzymes were used. In the *SspI* digestion, a three-band profile of 449, 267, and 108 bp was obtained in all experimental samples; this profile confirmed *Cryptosporidium* spp. infection at the genus level. In the *MboII* digestion, a 771 bp band profile specific to *C. parvum* was detected in all samples. No evidence of mixed infection was found in any of the experimental groups; it was determined that the sole causative agent of the infection was *C. parvum* (Supplementary Figure S7).

### 3.6. Sequencing

The BLAST analysis revealed that the 6 samples *C. parvum* sequences identified in this study exhibited nucleotide similarity ranging from 99.39 to 100%. This high similarity indicates the genetic homogeneity of the isolates used in the study. The obtained gene sequences were submitted to the EMBL/GenBank database and published under the accession numbers PX933065, PX933066, PX933067, PX933068, PX933070, and PX933072. BLAST analysis revealed that the *C. parvum* sequences identified in this study showed 99.88–100% similarity to *C. parvum* isolates registered in GenBank. It was determined that one of the isolates identified in the study showed 100% similarity with *C. parvum* samples of human (AB089290) and calf (MK426795) origin from different countries.

## 4. Discussion

Cryptosporidiosis is a zoonotic disease caused by protozoa of the genus *Cryptosporidium*, which results in significant economic losses, particularly in calves, lambs, and kids [46]. Currently, there is no approved and widely used effective vaccine for cryptosporidiosis, and the number of available treatment options remains limited [47]. In this study, the therapeutic efficacy of albendazole in neonatal calves with diarrhea caused by cryptosporidiosis was evaluated by comparing it with the effects of paromomycin alone as a reference drug and the combined effects of albendazole plus paromomycin.

From a clinical perspective, the fact that the suckling reflex remained strong on all measurement days and that the mental status remained normal in the experimental groups indicates that the clinical presentation in the selected calves was mild to moderate in severity. This finding serves as significant clinical evidence that the infected animals did not develop advanced complications such as sepsis, severe dehydration, or hypovolemic shock [35,48]. Regarding changes in fecal score, a decrease from score 2 to 1 was observed in the albendazole group in the days following the completion of treatment, whereas the same change was evident in the paromomycin group starting from day 1. Compared to Masood et al. [49], who reported that the administration of albendazole (10 mg/kg) in cattle experimentally infected with *C. parvum* resulted in clinical improvement starting from the 6th day, the higher dose (20 mg/kg) used in the present study did not result in an improvement in the fecal score, but it was observed to correlate with the oocyst load. The observation of improvement in fecal scores starting from day 1 in calves naturally infected with *C. parvum* in the paromomycin group is consistent with the findings of early clinical response in the comparative study of halofuginone and paromomycin by Aydogdu et al. [50].

When evaluating the changes in oocyst shedding, one of the main outcomes of the study, we first note that the fecal oocyst counts did not follow a normal distribution and that their standard deviations were disproportionately large relative to the arithmetic means; the data were therefore re-analysed and are reported as median, using the Friedman test to assess the effect of time within each group and the Kruskal–Wallis test to compare groups at each sampling day, rather than repeated-measures ANOVA on means. Within each of the three treatment groups, oocyst counts declined significantly over the course of the study (*p* < 0.001), confirming that all three regimens were associated with a clear reduction in oocyst shedding over time. Descriptively, all three groups showed a marked reduction in median oocyst counts between day 1 and day 3, with the paromomycin and albendazole + paromomycin groups declining somewhat earlier than the albendazole group. However, the Kruskal–Wallis comparisons among the three treatment groups at day 1, 3 and 7 did not reach statistical significance. This indicates that, although the paromomycin and albendazole + paromomycin groups showed a numerically earlier decline in median oocyst counts, this apparent difference in the speed of oocyst clearance between albendazole and the other regimens should be regarded as a descriptive trend rather than a statistically confirmed difference, and it should be interpreted with caution given the wide ranges observed within each group.

With this caveat in mind, the numerically slower descriptive decline observed in the albendazole group is nonetheless compatible with albendazole’s mechanism of action. As a benzimidazole derivative, albendazole blocks the polymerization of tubulin dimers by interacting with the colchicine-binding site of β-tubulin in the microtubular system of *Cryptosporidium* spp. [26,27,51]. This inhibition halts cell division by preventing the formation of the mitotic spindle in parasite cells; it also leads to cell death by inhibiting the parasite’s glucose uptake and intracellular transport, thereby disrupting energy metabolism. For this effect to fully manifest, the drug must reach the actively dividing stages of the parasite. The transition to the intensive proliferation phase in the life cycle of *Cryptosporidium*, however, takes a certain amount of time. Therefore, the absence of a noticeable decrease in oocyst levels during the first few days of drug administration is a biochemically expected and consistent finding. Masood et al. [49] reported that administration of albendazole at doses of 7.5 mg/kg and 10 mg/kg resulted in a reduction in oocyst levels ranging from 34.8% to 64.6% between days 13 and 27 post-treatment. The use of a 20 mg/kg dose in the present study resulted in a similar reduction appearing much earlier (by days 3–7), which was interpreted as supporting the dose-response relationship and the ability of the higher dose to more rapidly exceed the tubulin inhibition threshold. In the paromomycin group, the median oocyst count had already fallen sharply by day 3. As an aminoglycoside antibiotic, paromomycin has limited systemic absorption and reaches high concentrations in the intestinal lumen following oral administration. Its mechanism of action involves irreversibly binding to the 30S subunit of the parasite’s prokaryote-like ribosomes, causing misreading of mRNA and consequently leading to defective protein synthesis. This ribosomal targeting, combined with the drug’s ability to create locally high concentrations in the intestinal lumen, results in a much more immediate and rapid antiparasitic effect against the parasite. This finding is consistent with the results reported by Fayer and Ellis [52] and Grinberg et al. [53], who found that paromomycin sulfate suppresses oocyst shedding in the early stages.

Live weight data clearly demonstrate the negative impact of cryptosporidiosis on growth performance. Healthy control calves achieved statistically significantly higher live weight values compared to the treatment groups throughout the entire observation period. Because control calves were healthy and non-diarrhoeic at enrolment, this difference in starting weight is expected and does not confound the assessment of growth, which was analysed as the within-animal percentage change from day 1. When percentage increases were compared to day 30, the treatment groups were found to have closed the gap with the control group. When comparing the treatment groups, the albendazole group showed higher live weight gain rates during the clinical examination period: +7.1% in the first 5 days, +16.4% at 10 days, and 57.3% at 30 days. These findings suggest that albendazole contributes positively to growth by suppressing the oocyst burden. A study by Shaw et al. [54] reported that calves that had severe cryptosporidiosis during the neonatal period had an average live weight 34 kg lower at 6 months compared to calves that showed no clinical signs. In the present study, it was determined that calves treated with albendazole achieved higher average live weights at both the 30-day mark and overall compared to other treatment groups. This suggests that albendazole may partially compensate for live weight loss in calves infected with *Cryptosporidium* spp. by alleviating clinical symptoms through a reduction in oocyst load.

When hematological parameters were evaluated, the fact that WBC, NEU, and LYM levels did not differ between groups indicates that the treatment protocols administered did not result in a significant difference in the leukocyte response. However, the fact that MON levels were statistically the highest in the paromomycin group (*p*_group_ = 0.019) can be interpreted as a reflection of a MON-induced tissue defense response accompanying a more rapid reduction in oocyst levels in this group. The fact that EOS values were higher in the control group compared to the treatment groups suggests that the stress response occurring during the acute phase of cryptosporidiosis infection may increase cortisol release, thereby removing eosinophils from circulation, and that eosinophil mobilization is suppressed in infected calves. When EOS levels were evaluated, although no statistically significant difference was detected between the groups (*p*_group_ = 0.525), an examination of the overall averages revealed that the control group had higher levels compared to the treatment groups, while the albendazole group exhibited the lowest values. When evaluated by measurement day, EOS values were found to rise significantly on days 20 and 30 in all groups, while they were at their lowest on day 7. Importantly, EOS values in the treatment groups were found to be higher than those in the control group on day 30. This situation may indicate that suppressed EOS mobilization recovers in the later stages of treatment as the infection is controlled and the oocyst load is reduced. Indeed, Sahu and Maiti [55] reported the observation of eosinophilia in calves infected with *Cryptosporidium*, and this finding was associated with defense mechanisms against the infection.

In red blood cell parameters, it is considered significant that MCHC was highest in the paromomycin group (*p*_group_ < 0.001), and RDW-CV and RDW-SD were highest in the albendazole + paromomycin group (*p*_group_ = 0.006 and 0.010). The increase in RDW is associated with pro-inflammatory cytokines disrupting erythrocyte maturation in infection and sepsis processes [56,57], and the regression of both parameters on day 30 can be considered an indicator of reduced inflammatory load during the recovery process. These findings are consistent with Uzlu et al. [58] and Mullakaev et al. [59], who reported that hematological changes in neonatal diarrheal calves can reflect both the severity of the disease and the dynamics of recovery.

Notably, significant time effects were also observed for blood parameters in the untreated healthy control group. This baseline drift is not treatment-related but reflects the well-documented ontogeny of the neonatal calf: erythrocyte indices, white blood cell parameter counts and serum analytes change markedly and predictably over the first month of life [44,45]. GGT, in particular, is high at birth owing to intestinal absorption of colostral GGT and declines physiologically thereafter [44]. Because sampling volumes were small (2 mL EDTA), standardised and taken at identical intervals in all groups, and because the same maturational trends were observed in the untreated control group, the fluctuations observed across timepoints are better explained by normal neonatal development and by infection-recovery dynamics than by an artefact of repeated venipuncture.

The most significant intergroup difference among biochemical parameters was observed at the ALB level (*p*_group_ < 0.001). The highest ALB values were measured in the control group and the lowest in the paromomycin group, and the lowest levels were recorded between days 3 and 7 in all treatment groups. This can be directly related to the villi atrophy and increased permeability caused by *C. parvum* in the intestinal mucosa, leading to protein loss [60]. This is consistent with the findings of Gharieb et al. [61], which showed a significant decrease in TP and ALB levels in diarrheal calves. The fact that GGT activity was high on day 0 in all groups and gradually decreased in the following days is consistent with the findings of Egli and Blum [44] and Kaçar et al. [62], which show that GGT taken with colostrum follows a decreasing physiological course over time. The higher AST levels measured in the control group compared to the treatment groups, peaking on day 30, reflect physiological changes related to enzyme activation in the intestines and hepatic adaptation processes during the first week of life [63]. The fact that CREAT, BUN, and UREA levels in the albendazole group remained within reference limits and no clinically significant changes were observed compared to the control group (*p*_group_ = 0.406 and *p*_measurement_ = 0.197) is a significant indication that 5 days of albendazole administration at a dose of 20 mg/kg maintained its renal and hepatic safety profile. This finding reveals that albendazole offers a significant advantage in terms of safety compared to the potential nephrotoxic risk reported for paromomycin, an aminoglycoside [33].

In this study, nested PCR and RFLP analysis of neonatal diarrheal calves with naturally occurring cryptosporidiosis confirmed *C. parvum* as the causative species at the molecular level in all study groups. The 99.88–100% similarity of the obtained sequences to human (AB089290) and calf (MK426795) isolates in GenBank clearly demonstrates the zoonotic potential of these strains. This finding is consistent with studies highlighting the prevalence and zoonotic importance of *C. parvum* in unweaned neonatal calves [46,64]. Studies conducted in different provinces of Türkiye have reported *Cryptosporidium* prevalence in diarrheal calves ranging from 7% to 63.3% [18,65,66]. In this study, the epidemiological data clearly demonstrate that infection with *C. parvum* in neonatal calves is a widespread and significant zoonotic disease-causing enteritis.

When the effectiveness of carbol fuchsin staining and immunochromatographic rapid diagnostic kits used in the diagnosis of cryptosporidiosis was evaluated in the treatment process, it was observed that both methods yielded largely consistent results in monitoring post-treatment oocyst shedding. In the albendazole group, microscopic examination and the rapid diagnostic kit detected positivity on days 3 and 7 in most patients, while one calf yielded negative results on day 3 with both methods. This is thought to be due to the oocyst shedding falling below microscopically detectable levels from day 3 onwards in that case. Indeed, parasite DNA was detected in the same sample on day 7 using nested PCR. In the paromomycin group, oocysts were not detected in six calves by microscopic examination and the rapid diagnostic kit after treatment, and oocysts were not detected after day 7 in five calves and after day 10 in one calf. This finding is consistent with the findings of Fayer and Ellis [52] and Grinberg et al. [53], which indicated that paromomycin suppresses oocyst shedding early. In the albendazole + paromomycin group, oocysts were not detected by either method in four calves from the start of treatment, in six calves after day 3, and in two calves after day 7. The parallel results of carbol fuchsin staining and the immunochromatographic kit demonstrate that the rapid diagnostic kit offers equivalent reliability to microscopy as a practical alternative in the field. Indeed, Chartier et al. [67] reported that immunological-based rapid tests exhibited comparable sensitivity to microscopic methods when oocyst density was above a certain threshold. However, it should be considered that both conventional methods are insufficient in detecting low oocyst loads and PCR can detect parasite DNA even in cases where oocyst shedding appears to have stopped.

## 5. Conclusions

When the findings of this study are evaluated as a whole, it is seen that oral albendazole administration at a dose of 20 mg/kg for 5 days effectively suppresses oocyst shedding in neonatal calf diarrhea caused by *C. parvum*, contributes to clinical improvement, and maintains a renal-hepatic safety profile. Although paromomycin alone provided a faster oocyst reduction, both drugs exhibited similar ultimate efficacy in terms of oocyst undetectable by day 10. The albendazole + paromomycin combination conferred no additional benefit over paromomycin therapy, and the present data do not support a synergistic interaction. Considering albendazole’s low cost, widespread availability and broad antiparasitic spectrum, it is considered that this drug offers a viable alternative treatment option, especially in herd management conditions where access to halofuginone and paromomycin is limited.

## Figures and Tables

**Table 1:** Clinical assessment of the sucking reflex, mental status, and stool consistency based on the scoring system.

Parameters	0	1	2	3
Sucking reflex	None	Weak	Middle	Normal/Strong
Mental status	Normal	Alert (mild depression)	Depressed (moderate depression)	Comatose (severe depression)
Fecal scoring	Normal, well-formed stool	Close to normal, paste-like, pudding-like	Loose, unformed stool, with a yogurt-like consistency	Watery, foul-smelling, diarrhea

**Table 2:** Live weight values and percentage change measurements compared to day 1.

Measurements ($\overset{-}{X}$ ± S)					
Group	Day 1 (kg)	Day 5 (kg) [%Δ]	Day 10 (kg) [%Δ]	Day 30 (kg) [%Δ]	General
Control	47.02 ± 4.78	53.62 ± 4.81 [+%14.0]	57.35 ± 4.82 [+%22.0]	71.29 ± 5.36 [+%51.6]	57.32 ± 10.16 ^A^
Albendazol	39.85 ± 3.81	42.68 ± 2.98 [+%7.1]	46.37 ± 2.89 [+%16.4]	62.71 ± 4.32 [+%57.3]	47.01 ± 11.66 ^B^
Paromomycin	37.60 ± 5.65	39.85 ± 5.45 [+%6.0]	43.57 ± 5.61 [+%15.9]	59.97 ± 7.26 [+%59.5]	44.00 ± 12.20 ^B^
Albendazol + Paromomycin	38.88 ± 4.91	41.42 ± 4.30 [+%6.5]	44.98 ± 4.21 [+%15.7]	58.62 ± 4.58 [+%50.8]	45.98 ± 8.84 ^B^
General	40.94 ± 5.97 ^d^	44.54 ± 7.02 ^c^	48.26 ± 7.10 ^b^	63.37 ± 7.28 ^a^	48.72 ± 11.92

$\overset{-}{X}$: arithmetic mean; S: standard deviation; A, B: statistical difference between groups (*p* < 0.05); a, b, c, d: statistical difference between measurement days; [%Δ]: percentage change relative to the reference measurement (Day 1) for each group; *p*_group_ < 0.001; *p*_measurement_ < 0.001; *p*_group × measurement_ < 0.001.

**Table 3:** Comparison of immunochromatographic test kit results among experimental groups.

Group	No. of Animals	Day 0	Day 3	Day 7	Day 10	Day 20	Day 30
Control	0	0 *	0 *	0 *	0 *	0 *	0 *
Albendazol	12	12 *	12 *	6 *	0 *	0 *	0 *
Paromomycin	12	12 *	6 *	1 *	0 *	0 *	0 *
Albendazol + Paromomycin	12	12 *	8 *	2 *	0 *	0 *	0 *

* Number of confirmed cases.

**Table 4:** Comparison of carbol fuchsin staining results among experimental groups.

Group	No. of Animals	Day 0	Day 3	Day 7	Day 10	Day 20	Day 30
Control	0	0 *	0 *	0 *	0 *	0 *	0 *
Albendazol	12	12 *	12 *	6 *	0 *	0 *	0 *
Paromomycin	12	12 *	6 *	1 *	0 *	0 *	0 *
Albendazol + Paromomycin	12	12 *	8 *	2 *	0 *	0 *	0 *

* Number of confirmed cases.

**Table 5:** Changes in the number of *Cryptosporidium* oocysts per gram of feces by treatment group and day.

Measurements (×10^6^)					
Group	Day 1 (×10^6^)	Day 3 (×10^6^)	Day 7 (×10^6^)	Day 10 (×10^6^)	*p* *
Albendazol	65 (50–120)	20 (4–132)	0 (0–6)	0 (0–0)	χ^2^ = 56.339, df = 5, *p* < 0.001
Paromomycin	60 (27–204)	0 (0–14)	0 (0–0)	0 (0–0)	χ^2^ = 50.703, df = 5, *p* < 0.001
Albendazol + Paromomycin	91 (55–265)	3 (0–35)	0 (0–0)	0 (0–0)	χ^2^ = 46.621, df = 5, *p* < 0.001
p **	χ^2^ = 0.487, df = 2, *p* = 0.784	χ^2^ = 4.925, df = 2, *p* = 0.085	χ^2^ = 4.434, df = 2, *p* = 0.109	NA	

*p* *: within-group effect of time (Friedman test). *p* **: between-group comparison at each day (Kruskal–Wallis test). NA: not applicable (all groups reached a median of zero).

## Data Availability

The data presented in this study are available on request from the corresponding author due to privacy restrictions.

## Supplementary Materials

The following supporting information can be downloaded at https://www.mdpi.com/article/10.3390/pathogens15080854/s1. Figure S1: Line graph of WBC, NEU, LYM, MON and EOS parameters.; Figure S2: Line graph of red blood cell parameters of RBC, HGB, HCT and MCV parameters.; Figure S3: Line graph of red blood cell parameters of MCH, MCHC, RDW-CV and RDW-SD parameters.; Figure S4: Line graph of serum biochemistry parameters of TP, UREA, GGT and BUN parameters.; Figure S5: Line graph of serum biochemistry parameters of ALB, AST and CREAT parameters.; Figure S6: Line graph of PLT, MPV, PDW and PCT parameters.; Figure S7: RFLP analysis results of the treatment groups; M: marker; N: negative control (genomic DNA from non-infected feces); P: positive control (*C. parvum*); lanes 1–7 (Group 2), *Cryptosporidium* sp.; lanes 8–13 (Group 3), *Cryptosporidium* sp.; lanes 14–19 (Group 4), *Cryptosporidium* sp. (A) RFLP results obtained with *SspI* digestion. (B) RFLP results obtained with *MboII* digestion.

## Abbreviations

The following abbreviations are used in this manuscript:

ALB	Albumin
AST	Aspartate aminotransferase
BLAST	Basic local alignment search tool
BRSV	Bovine respiratory syncytial virus
BUN	Blood urea nitrogen
CBC	Complete blood count
CREAT	Creatinine
EOS	Eosinophils
GGT	Gamma-glutamyl transferase
HCT	Hematocrit
HGB	Hemoglobin
LYM	Lymphocyte
MCH	Mean corpuscular hemoglobin
MCHC	Mean corpuscular hemoglobin concentration
MCV	Mean corpuscular volume
MON	Monocyte
MPV	Mean platelet volume
NCBI	National center for biotechnology information
NEU	Neutrophil
PCR	Polymerase chain reaction
PCT	Plateletcrit
PDW	Platelet distribution width
PI3	Parainfluenza type 3
PLT	Platelet
RBC	Red blood cell
RDW-CV	Red blood cell distribution width-coefficient of variation
RDW-SD	Red blood cell distribution width -standard deviation
RFLP	Restriction fragment length polymorphism
TP	Total protein
UREA	Urea
WBC	White blood cell
